# Consequences of Implementing Neutralizing Buffered Peptone Water in Commercial Poultry Processing on the Microbiota of Whole Bird Carcass Rinses and the Subsequent Microbiological Analyses

**DOI:** 10.3389/fmicb.2022.813461

**Published:** 2022-03-17

**Authors:** Jennifer A. Wages, Dana K. Dittoe, Kristina M. Feye, Steven C. Ricke

**Affiliations:** ^1^Cell and Molecular Biology, University of Arkansas, Fayetteville, AR, United States; ^2^Tyson Foods, Inc., Springdale, AR, United States; ^3^Meat Science and Animal Biologics Discovery Program, Animal and Dairy Sciences Department, University of Wisconsin-Madison, Madison, WI, United States

**Keywords:** neutralizing Buffered Peptone Water, whole bird carcass rinsates, microbiome, poultry (chicken), *Campylobacter*

## Abstract

In 2016, the United States Department of Agriculture (USDA) Food Safety and Inspection Service (FSIS) established guidelines which modified the Buffered Peptone Water (BPW) rinsate material to include additional compounds that would better neutralize residual processing aids and allow for better recovery of sublethal injured *Salmonella* spp. cells. While the added compounds improved the recovery of *Salmonella* spp., specific data to understand how the new rinse agent, neutralizing Buffered Peptone Water (nBPW), impacts the recovery of other microorganisms such as *Campylobacter* spp. and indicator microorganisms are lacking. Therefore, this study evaluated the impact of rinse solutions (BPW or nBPW) used in Whole Bird Carcass rinsate (WBCR) collections on the subsequent microbiome and downstream culturing methodologies. Carcasses exiting a finishing chiller were rinsed in 400 ml of BPW or nBPW. Resulting rinsates were analyzed for Enterobacteriaceae (EB), *Salmonella*, and *Campylobacter* spp. prevalence and total aerobic bacteria (APC) and EB load. The 16S rDNA of the rinsates and the matrices collected from applied microbiological analyses were sequenced on an Illumina MiSeq^®^. Log_10_-transformed counts were analyzed in JMP 15 using ANOVA with means separated using Tukey’s HSD, and prevalence data were analyzed using Pearson’s χ^2^ (*P* ≤ 0.05). Diversity and microbiota compositions (ANCOM) were analyzed in QIIME 2.2019.7 (*P* ≤ 0.05; *Q* ≤ 0.05). There was an effect of rinsate type on the APC load and *Campylobacter* spp. prevalence (*P* < 0.05), but not the quantity or prevalence of EB or *Salmonella* spp. prevalence. There were differences between the microbial diversity of the two rinsate types and downstream analyses (*P* < 0.05). Additionally, several taxa, including *Streptococcus*, *Lactobacillus*, *Aeromonas*, *Acinetobacter*, *Clostridium*, Enterococcaceae, Burkholderiaceae, and Staphylococcaceae, were differentially abundant in paired populations. Therefore, the rinse buffer used in a WBCR collection causes proportional shifts in the microbiota, which can lead to differences in results obtained from cultured microbial populations.

## Introduction

Verification programs associated with poultry processing often use targeted microbiological analyses to determine levels of pathogens and indicator microorganisms to ascertain system performance. The performance of the system in terms of mitigating and controlling pathogens in final products is of upmost importance to both the industry and the United States Department of Agriculture’s Food Safety Inspection Service (USDA–FSIS) as raw poultry carcasses and parts are commonly implicated as contributing sources of foodborne disease caused by *Salmonella* and *Campylobacter* (Tompkin, 1990; Bryan and Doyle, 1995; Painter et al., 2013; Antunes et al., 2016; Skarp et al., 2016).

Human pathogens, which are often commensal within the gastrointestinal tract (GIT) of broilers, are often liberated from the feathers, feet, crop, and GIT during commercial poultry processing (Josefsen et al., 2015; Roccato et al., 2018). To aid the control of human pathogens, processing facilities and abattoirs limit the amount of contamination and cross-contamination events using multi-hurdle interventions that commonly include carcass and equipment washes, product chilling, and the application of antimicrobials (Stopforth et al., 2007; Wideman et al., 2016; Dogan et al., 2019). To measure the antimicrobial efficacy of applied control steps, whole bird carcass rinsates (WBCRs) are collected at sites that represent pre-intervention and post-intervention locations within the processing system and evaluated for target pathogenic microorganisms as well as indicator and spoilage microorganisms (Saini et al., 2011; Milios et al., 2014). The microbiological evaluations of these rinses provide quantifiable data that can be used to assess the performance of specific processing steps and interventions within a poultry harvest system (Stopforth et al., 2007; Milios et al., 2014).

As BPW provides a non-selective nutrient source with a built-in phosphate buffering system which helps maintain a neutral pH level, BPW has been widely used to aid in the recovery of *Salmonella* spp. from collected samples (Baylis et al., 2000). However, as the industry has evolved and introduced new interventions and post-chill applications, research by Gamble et al. (2016) indicated that the use of BPW alone may not be wholly suitable to mitigate the antimicrobial carryover that may occur during WBCR sample collection. Furthermore, the presence of a non-neutralized antimicrobial in the rinsate could hinder the recovery of some microorganisms, namely, *Salmonella* spp., from the collected rinsates and lead to false-negative results (Gamble et al., 2016). In response to the identified limitations of BPW as a resuscitative medium, in 2016, the USDA–FSIS began using a specialized neutralizing Buffered Peptone Water (nBPW) to collect whole bird carcass (WBC). The modified BPW solution included sodium thiosulfate (1.0 g/1.0 L), sodium bicarbonate (12.5 g/1.0 L), and soy lecithin (7.0 g/1.0 L) to provide more effective neutralization of a broader spectrum of antimicrobials and further aid in the recovery of *Salmonella* from collected rinsates (Gamble et al., 2017). Specifically, the addition of sodium thiosulfate aids in the neutralizing antimicrobial properties of chlorine and iodine; sodium bicarbonate increases the acid-neutralizing capacity of the solution; and soy lecithin neutralizes the effects of quaternary ammonia compounds.

While the application of nBPW had been identified as aiding in the recovery of *Salmonella* spp. from poultry processing samples, the application of these materials to address the recovery of *Campylobacter* spp. had not been specifically evaluated prior to the introduction of the new rinsate. Additionally, the same materials are often used by processors and researchers to collect samples related to investigations of process inefficiencies, which typically include analysis of the rinsates for both *Salmonella* spp. and *Campylobacter* spp. as well as indicator organisms, such as total aerobic bacteria (APC), Enterobacteriaceae (EB), and *Escherichia coli* (Bourassa et al., 2019, Yu et al., 2019). The information obtained from enumeration and indicator organisms are a necessity to monitor both sanitary protocol measures and applied process controls, as the target pathogens (*Salmonella* and *Campylobacter* spp.) may be present at low levels and not necessarily homogenously dispersed within the process system (Bourassa et al., 2019; Yu et al., 2019).

**FIGURE 1:**
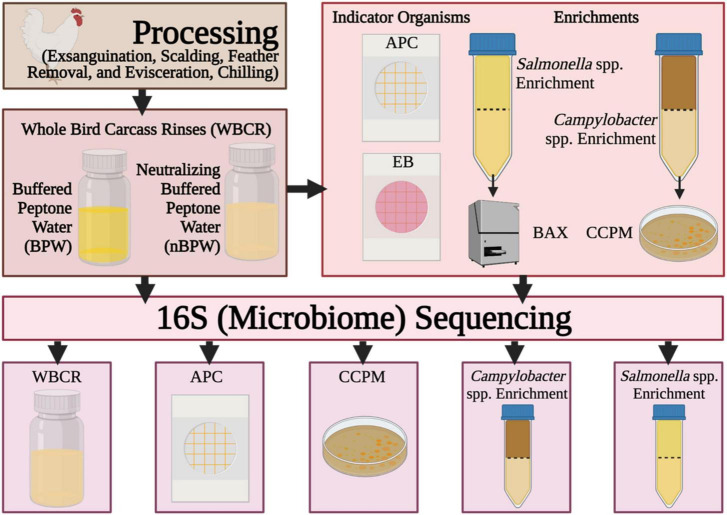
Flow diagram of the procedures and samples collected during the experiment. Created with BioRender.com.

In this study, 16S rDNA sequencing was performed to better understand the effect of the compositions of the two rinse solutions, BPW and nBPW, on the recovery of *Salmonella* spp., *Campylobacter* spp., and indicator microorganisms. Specifically, compositional analysis of the microbiota recovered in the BPW and nBPW rinsates that were collected after carcasses exited a finishing chiller equipped with peroxyacetic acid (PAA) at a commercial broiler processing facility was evaluated (Figure 1). Additionally, the microbiota present within *Salmonella* spp. and *Campylobacter* spp. enrichment broths, as well as bacterial growth from *Campylobacter jejuni*/*C. coli* chromogenic plating medium (CCPM) agar used to ascertain *Campylobacter* spp. prevalence and corresponding 3M™ Petrifilm™ used to obtain indicator counts of total APC and Enterobacteriaceae (EB) were compared. Elucidating the microbiota compositions of the different rinsates and materials from associated culturing methodologies provides an improved understanding of how the rinsate media composition alters the results of microbiological analyses.

## Materials and Methods

### Sample Collection

Sample collection occurred within a USDA-inspected, commercial facility, processing approximately 240 Cornish hens per minute using two evisceration lines which fed into one immersion chilling system. As these samples were collected at a USDA-inspected, commercial facility, during normal operation, this project was exempt from the University of Arkansas Institutional Animal Care and Use Committee (IACUC) approval. Samples were collected after exiting a 20–30 s finishing chiller containing 800–1,000 ppm PAA. To ensure that the carcasses collected were representative of the same flock and subjected to similar processing conditions, all collections occurred in rapid succession. Eighty total WBCRs were collected, and the first and last pairs of samples were collected within 1.5 h of each other.

Carcasses representing two evisceration lines, but one chilling system, were collected in pairs as carcasses were deposited on the conveyor system. New, sterile gloves were used to collect each pair, and carcasses were collected with gloved hands, one carcass in each hand, and hung by legs on shackles and allowed to drip for 1 min. After drip time elapsed, the carcasses were placed in sterile rinse bags (Fisher Scientific, Pittsburgh, PA, United States). For each pair of WBC collected, 400 ml of either nBPW (CultureMediaConcepts, Millennium LifeSciences, Inc., Anaheim, CA, United States) or BPW (Becton, Dickinson and Company, Sparks, MD, United States) was poured over the surface and interior cavity of the individual carcass. The base ingredients of both solutions contain peptone (10.0 g/1.0 L), sodium chloride (5.0 g/1.0 L), disodium phosphate (3.5 g/1.0 L), and potassium dihydrogen phosphate (1.5 g/1.0 L), but additionally, the nBPW solution also includes sodium thiosulfate (1.0 g/1.0 L), sodium bicarbonate (12.5 g/1.0 L), and soy lecithin (7.0 g/1.0 L) to provide more effective neutralization of a broader spectrum of antimicrobials. After addition of either BPW or nBPW, following USDA-FSIS (2021) collection practices, the carcasses were rinsed for 1 min in a 90° arcing motion to ensure that the rinsate traversed along the carcass surface and cavity. At the end of the 1-min rinse, the rinsate was subsequently transferred to the original container, and the carcass was returned to the processing line. After collection, all rinses were placed on ice in a cooler and transported to the testing laboratory for same-day analysis.

Upon arrival at the laboratory, the pH level of five subsamples of each type of rinsate was determined using pH indicator strips. In brief, samples were swirled to mix, and then approximately 1.0 ml of the sample was aseptically removed and deposited onto a pH paper. The pH paper was compared to standard color range on packaging to determine the approximate pH level of the sample. The pH ranged from 6.0 to 6.5 among the BPW rinses and ranged between 7.0 and 7.5 among the nBPW rinses.

### Microbiological Analyses

#### Indicator Organisms

On the same day of collection, rinsates were enumerated for APC and EB with 3M™ APC Petrifilm™ and 3M™ Enterobacteriaceae Petrifilm™ (3M™, St. Paul, MN, United States) per Official Methods of Analysis (OMA) 990.12 and 2003.01, respectively, published by the International Association of Official Analytical Chemists (AOAC). In brief, a 1.0 ml aliquot of the homogenous original rinse material was 1:10 serially diluted in 9 ml of Butterfield’s phosphate buffer (Thermo Scientific, Remel, Lenexa, KS, United States) to 10^–6^. The prepared serial dilutions were pipetted onto the surface of APC and EB Petrifilm™, which includes both gelling and nutrient agents for the detection of APC or EB microorganisms, dependent on the class of Petrifilm™ used. Incubation and subsequent enumeration of Petrifilm™ occurred per prescribed published protocol except for the incubation temperature of the EB Petrifilm, which was altered to align with USDA–FSIS Microbiological Laboratory Guidebook (MLG) Chapter 3.02 (USDA-FSIS, 2015), in which EB detection for meat and poultry products are typically incubated at 35 ± 1°C. In addition to EB count data, the presence or absence of EB typical growth from direct plating analysis was used to determine EB prevalence within the sample sets.

#### *Campylobacter* spp. Detection and Quantification

To determine the prevalence of *Campylobacter* spp., 30 ml aliquots of the homogenous original rinsate samples were enriched in 30 ml of 2X blood-free Bolton broth (Thermo Fisher, Oxoid Ltd., Basingstoke, Hampshire, United Kingdom). The 2X blood-free Bolton broth was made according to USDA–FSIS standards (MLG 41.04; USDA-FSIS, 2016) and consisted of two times the concentration of both the broth and antibiotics without the inclusion of lysed horse blood. The modification of the Bolton broth is critical for the enrichment of the rinsates so that there are enough selective nutrients and antibiotics for the final volume: 60 ml of enrichment (USDA-FSIS, 2016). The enrichments were incubated at 42 ± 1°C under microaerophilic conditions (85% nitrogen, 10% carbon dioxide, and 5% oxygen) for 48 ± 2 h per USDA–FSIS guidelines (USDA-FSIS, 2016). Approximately, 40 ml of the enrichment was aliquoted to a sterile 50 ml conical tube for 16S rDNA sequencing.

After incubation, the prevalence of *Campylobacter* spp. was determined by inoculating an R&F^®^ CCPM (R&F Products, Downers Grove, IL, United States) agar plate with 10 μl of incubated sample. Inoculated plates were incubated at 42 ± 1°C under microaerophilic conditions per manufacturer recommendations. After 48 ± 2 h of incubation, plates were evaluated for the presence of typical *Campylobacter* spp. colonies, which appear pink or salmon in color. The plates were wrapped in Parafilm^®^ M (Bemis Company, Inc., Neenah, WI, United States) and held between 2 and 8°C until the surface colonies could be harvested from the plates for 16S rDNA sequencing.

#### *Salmonella* spp. Detection and Quantification

To determine the prevalence of *Salmonella*, 30 ml aliquots of the original rinsates were enriched in 30 ml of BPW (Becton, Dickinson and Company, Sparks, MD, United States) regardless of the original buffer composition used in the collection of the WBCR (BPW or nBPW). The enrichments were incubated at 35 ± 1°C for 20–24 h. Approximately 5 μl of the *Salmonella* enrichments was analyzed for *Salmonella* prevalence utilizing Hygiena© BAX^®^ (Hygiena, LLC, Camarillo, CA, United States) PCR-based testing system and the Hygiena© BAX^®^ *Salmonella* assay kit per guidelines provided in OMA 2003.09 AOAC. The 5 μl of the *Salmonella* enrichments was added to cluster tubes containing 200 μl of the provided lysis reagent (1 μl of protease to 80 μl of lysis buffer) and lysed by heating the tubes in a heating block set at 37°C for 20 min, at 95°C for 10 min, and at 2°C to 8°C for at least 5 min. Lastly, 30 μl of the lysate was added directly to the dehydrated PCR tablets and allowed to incubate at 2 to 8°C for 10–30 min. The rehydrated PCR tablets were placed in the BAX^®^ where the genomic DNA of *Salmonella* was amplified. Qualitative results were recorded and exported for statistical analysis. For 16S rDNA sequencing, 40 ml of the enrichment was aliquoted to a sterile 50 ml conical tube and immediately processed.

### DNA Extraction and Library Preparation

#### DNA Extraction From Rinsates and Enrichments

Upon reaching the testing laboratory, aliquots of the collected 400 ml rinsates were prepared for DNA extraction by aliquoting a total of 80 ml into two sterile 50 ml conical tubes and centrifuged (Sorvall Lynx 6000 with BIOFlex HS rotor, Thermo Fisher Scientific, Langenselbold, Germany) for 15 min at 8,000 × *g*. Also, 40 ml aliquots of the *Salmonella* spp. and *Campylobacter* spp. incubated enrichments was centrifuged for 15 min at 8,000 × *g* to achieve initial pellet material. The supernatants were decanted, and the pellets were resuspended in room temperature 1 × PBS (Oxoid Microbiology Products, Thermo Scientific, Wilmington, DE, United States) at two times the volume of the pellet and centrifuged for an additional 15 min at 8,000 × *g* (resuspended in 1–2 ml of 1 × PBS). This wash step was completed twice, and the final washed pellet was resuspended in 500 μl of 1 × PBS and stored at −80°C until the genomic DNA could be extracted. The genomic DNA of the resuspended pelleted material from the rinsates and the *Salmonella* spp. and *Campylobacter* spp. incubated enrichments were extracted using a QIAamp DNA Stool Mini Kit (Qiagen, Valencia, CA, United States). In brief, 1.0 ml of supplied Inhibitex buffer was added to the 500 μl resuspended pellets and vortexed continuously for a minute or until the sample appeared thoroughly homogenized. The resulting mixture was heated for 5 min at 70°C to lyse cells, and the subsequent lysate was vortexed for 15 s and centrifuged for 1 min at 1,000 × *g* to pellet particles. Further steps were performed as indicated in the manufacturer’s standard protocol except for doubling the volume of proteinase K used and using DNase/RNase-free water (Fisher Scientific) at a reduced volume of 30 μl for the final eluent.

#### DNA Extraction From Petrifilm™ Plates

Prior to extraction, the APC and EB Petrifilm™ plates were held at −20°C to limit further changes in bacterial growth. With a sterile loop, the gel matrix and colonies (between 5 and 500 colonies from APC and 0 to 100 from EB) of the Petrifilm™ were harvested into a 2 ml microcentrifuge containing 200 μl of 1 × PBS. The samples were resuspended by vortexing and subsequently centrifuging at 8,000 × *g* for 1 min. The supernatants were discarded, and the pellets were resuspended in 500 μl of 1 × PBS and centrifuging at 8,000 × *g* for 1 min. Next, the DNeasy Blood and Tissue Kit (Qiagen) pretreatment protocol for Gram-positive cells was used with some modification (Kim et al., 2017). Pellets were resuspended in 400 μl of enzymatic lysis buffer (20 mM Tris-Cl, 2 mM EDTA, 1.2% Triton™ X-100, and 20 mg/ml of lysozyme; Dow Chemical Company, Midland, MI, United States), vortexed for 20 s, and incubated at 37°C for 60 min. Proteinase K (25 μl) was added to the subsequent suspension and vortexed, and 200 μl of Buffer AL (without ethanol) was added to the lysate. The lysate was further treated by incubating in a water bath for 30 min at 56°C and subsequently vortexed at 8,000 × *g* for 3 min. The supernatant (800 μl) was added to a fresh microcentrifuge tube, and 200 μl of molecular-grade ethanol (Decon Labs, Inc., King of Prussia, PA, United States) was added and homogenized thoroughly. The remainder of the extraction procedure followed the standard protocol without deviation. Due to limited growth on the EB Petrifilm™ plates, low levels of genomic DNA were isolated and therefore precluded those samples from being further evaluated.

#### DNA Extraction From Agar Plates

The R & F^®^ CCPM plates used to evaluate *Campylobacter* spp. prevalence were held at refrigerated temperatures (2–8°C) for 7–10 days until the surface of the agar plate could be harvested. All colonies on the surface of the CCPM agar plates were harvested by sweeping a sterile loop across the entire surface of the agar plate (less than 10 colonies), and the collected material was deposited into a microcentrifuge containing 200 μl of 1 × PBS. The mixture was homogenized thoroughly and centrifuged at 17,000 × *g* for 1 min to pellet the material. The pellet was washed in 500 μl of 1 × PBS and resuspended in 200 μl of PBS. The genomic DNA of the subsequent suspension was extracted by employing the same methodology described to extract the genomic DNA of the Petrifilm™ samples using the Gram-positive protocol of the DNeasy Blood and Tissue Kit.

#### Library Preparation

The extracted genomic DNA was quantified using a NanoDrop 1000 (Thermo Fisher Scientific, Waltham, MA, United States). If the DNA concentration was above 15 ng/ml, the sample was diluted to 10 ng/ml in sterile nuclease-free water. Targeted 16S rDNA libraries from all samples were constructed by using dual-indexed primers developed by Kozich et al. (2013) and AccuPrime™ Pfx SuperMix (Thermo Scientific) to amplify the V4 region of the 16S rRNA gene sequences. The PCR amplification procedure consisted of the following protocol on an Eppendorf Mastercycler X50a (Eppendorf, Hamburg, Germany): initial denaturation at 95°C for 5 min and 35 cycles of 95°C for 15 s (denature), 55°C for 30 s (anneal), and 68°C for 1 min. The ramp speed for the current protocol was uniformly set at 1°C/s. The amplification of the targeted region of all samples and the non-amplification of the no template control were confirmed using gel electrophoresis. Exactly 18 μl of the confirmed amplified amplicons was normalized using a SequelPrep™ Normalization Plate Kit (Thermo Fisher Scientific, Waltham, MA, United States) to achieve an equimolar concentration and equal volume of the samples (∼0.8 ng/μl). Normalized samples were pooled by PCR plate, and subsequently, these pools were then combined to make a final library. The pooled plates and final library were quantified using a Qubit fluorometer (Thermo Fisher Scientific, Waltham, MA, United States) and quantitative PCR using the KAPA qPCR library quantification kit (Kapa Biosystems, Inc., Wilmington, MA, United States). An Agilent Bioanalyzer (Agilent, Santa Clara, CA, United States) was used to confirm amplicon size and that there was no significant presence of background DNA.

#### 16S Amplicon Sequencing

The concentration of the final library was converted to pM, denatured in 5 μl of 0.2 N NaOH for 5 min at room temperature, diluted to 20 pM in HT1 buffer, and further diluted to 6 pM in HT1 buffer. The PhiX v3 (Illumina, San Francisco, CA, United States) was also denatured in 5 μl of 0.2 N NaOH, diluted to 20 pM in HT1 buffer, and further diluted to 6 pM in HT1 buffer. The diluted library and PhiX were then combined for a final concentration of 30% PhiX and loaded into a MiSeq^®^ v2 (500-cycle) reagent cartridge (Illumina, San Francisco, CA, United States) per the manufacturer’s instructions. Custom forward and reverse primers and index (3 μl) developed by Kozich et al. (2013) were added directly to the wells containing the Illumina primers. The sequencing cartridge was then loaded into an Illumina MiSeq (Illumina, San Francisco, CA, United States). At the completion of the sequencing run, raw sequences were converted to demultiplexed sequences onboard the MiSeq, and subsequent fastq files were automatically uploaded into the BaseSpace Hub (Illumina, San Francisco, CA, United States). Data generated from this study are available at github.com (RickeLab-UW) and on the Short Read Archive at NCBI (BioProject: PRJNA765223).

### Bioinformatic and Statistical Analyses

#### Data Filtering and Taxonomic Analysis

Demultiplexed sequences were retrieved from BaseSpace Hub and uploaded into the Quantitative Insights into Microbial Ecology 2 (QIIME 2) pipeline version 2019.7 using Casava 1.8 (Bolyen et al., 2018). With the use of DADA2 (*via* q2-dada2), demultiplexed paired reads were denoised and trimmed for quality, with chimeras being filtered out *via* consensus filtering (Callahan et al., 2016). The resulting amplicon sequence variant (ASV) table was used for all downstream analyses, including taxonomic assignment, phylogenetic diversity measurements (alpha and beta), and differential abundance comparisons. A phylogenetic tree was generated using the q2-phylogeny plugin (Katoh et al., 2002; Katoh and Standley, 2013). Taxonomic assignment was performed using the trained classifier Silva 132 99% OTUs full-length sequences (MD5: 6a9aa92fc12f6e26d17df18b3e603417) with an applied confidence level of 95% (Bokulich et al., 2018; *via* q2-alignment).

#### Filtering Feature Table for Downstream Analyses

At the completion of taxonomic alignment, the feature table was filtered using metadata-based filtering in QIIME 2. The feature table was filtered according to the original samples (rinsate), non-selective and selective media (APC Petrifilm™ and CCPM agar plates), and enrichments (*Salmonella* spp. and *Campylobacter* spp. enrichments). The resulting five tables were used to elucidate the impact of rinsing buffer (BPW vs. nBPW) on the corresponding microbiota of the enrichments and selective media of subsequent WBCR (Figure 1).

#### Alpha and Beta Diversity Analyses

Initial alpha and beta diversity metric analyses were conducted using the q2-diversity plugin. The sampling depth of each feature table was chosen based on the lowest depth of subsequent samples. The quantitative metrics of evenness and richness, Pielou’s Evenness and Shannon diversity index, were used to determine the impact of the rinse type (nBPW vs. BPW) on the downstream alpha diversity of the original samples (rinsate), non-selective and selective media (APC Petrifilm™ and CCPM agar plates), and enrichments (*Salmonella* spp. and *Campylobacter* spp. enrichments). Qualitative metrics of richness were also evaluated using Observed Features and Faith’s Phylogenetic diversity, with the latter incorporating phylogenetic relationships of the sequences. The statistical significance of the impact of the buffer type (nBPW and BPW) used to perform a WBCR on the alpha diversity within the original samples (rinsate), non-selective and selective media (APC Petrifilm™ and CCPM agar plates), and enrichments (*Salmonella* spp. and *Campylobacter* spp. enrichments) was evaluated using the Kruskal–Wallis H test (Kruskal and Wallis, 1952). Beta metrics in the current study included the quantitative Bray–Curtis dissimilarity, qualitative Jaccard dissimilarity, and phylogenetic-based UniFrac (weighted and unweighted) (Lozupone et al., 2007). The impact of buffer type (nBPW and BPW) on the distance between the respective samples was determined using permutational multivariate analysis of variance (PERMANOVA), and the impact of buffer type on the dispersion of data was determined with permutational multivariate homogeneity of dispersion (PERMDISP). The main effects were considered significant when *P* ≤ 0.05, and the pairwise differences were *Q* ≤ 0.05. The *Q*-value represents the *P*-value as adjusted for a strict false discovery rate.

#### Compositional Analyses

The compositional analysis of the respective microbiomes (ANCOM) of the original samples (rinsate), non-selective and selective media (APC Petrifilm™ and CCPM agar plates), and enrichments (*Salmonella* spp. and *Campylobacter* spp. enrichments) was used to identify differentially abundant features within the paired populations of the two buffer types (nBPW and BPW) (Mandal et al., 2015). The sequences obtained from the *Campylobacter* spp. selective enrichments and CCPM agar plates used to determine prevalence and load of *Campylobacter* spp. were analyzed further. Further analysis removed sequences identified as *Campylobacter* and *Helicobacter* using taxonomy-based filtering of the feature tables in QIIME 2 to elucidate the remaining microbiota within these sample types. The rinsates, Petrifilm,™ and *Salmonella* spp. enrichment sample sets were analyzed using the complete feature tables (including *Campylobacter* spp.). The main effects were considered significant when *P* ≤ 0.05.

#### Analysis of Microbiological Enumeration and Enrichment

The detection limit of enumeration was determined to be less than 1 colony-forming unit (CFU) per milliliter. The CFUs from the duplicate plates of the selective media were averaged and log_10_ transformed prior to data analysis. Results that were less than 1 CFU/ml were recorded as 0.1 CFU/ml as log_10_ values of zero counts are undefined. With the use of SAS^©^ JMP^®^ 15.0 (SAS© Institute Inc., Cary, NC, United States), count data were analyzed using a one-way analysis of variance (ANOVA) with means separated using Tukey’s protected HSD. Prevalence data, a binomial response of positive (growth) or negative (no growth), were analyzed using Pearson’s χ^2^ analysis. Significance was determined at *P* ≤ 0.05.

## Results

### Difference in the Abundance and Prevalence of Organisms Between WBCR Buffer Types

**FIGURE 2:**
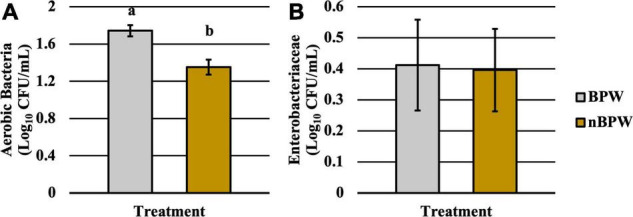
Impact of buffer type, nBPW and BPW, of WBCR on the **(A)** APC and **(B)** EB abundance (*N* = 80, *n* = 40, *k* = 2; *P* = 0.0002 and 0.9355, respectively). Gray bar denotes the BPW rinsates, whereas the yellow bar denotes nBPW rinsates. Values (ab) denote significant difference (*P* < 0.05).

**FIGURE 3:**
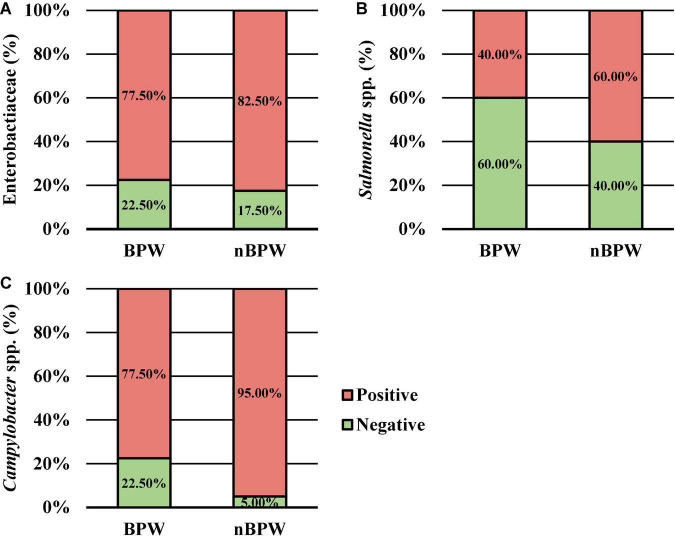
The impact of buffer type, nBPW and BPW, of WBCR on the prevalence of **(A)** Enterobacteriaceae, **(B)** *Salmonella* spp., and **(C)** *Campylobacter* spp. (*N* = 80, *n* = 40, *k* = 2; *P* = 0.5757, 0.0736, and 0.0231, respectively).

There was an effect of the buffer type (BPW vs. nBPW) on the abundance of APC enumerated from the APC Petrifilm™ (*P* < 0.05). The APC enumerated from the BPW rinsates were 0.39 log_10_ CFU/ml higher than that of the nBPW rinsates (Figure 2). In contrast, there was no difference between the abundance of Enterobacteriaceae (*P* > 0.05). The mean Enterobacteriaceae load was 0.41 and 0.40 log_10_ CFU/ml among the BPW and nBPW rinsate types, respectively. There was a main effect of rinsate type on the prevalence of *Campylobacter* spp. within the enrichments of the original rinsates with there being a higher prevalence of *Campylobacter* among the nBPW rinsates (95.00% positive) compared to BPW rinsates (77.50% positive) (Figure 3; *P* < 0.05). There was no effect of rinsate type on the prevalence of both *Salmonella* spp. and Enterobacteriaceae (*P* < 0.05), although the prevalence was numerically higher among the nBPW rinsates compared to the BPW rinsates (60.00 and 40.00% and 82.50 and 77.50%, respectively).

### Diversity Shifts and Corresponding Microbiota of WBCRs

**FIGURE 4:**
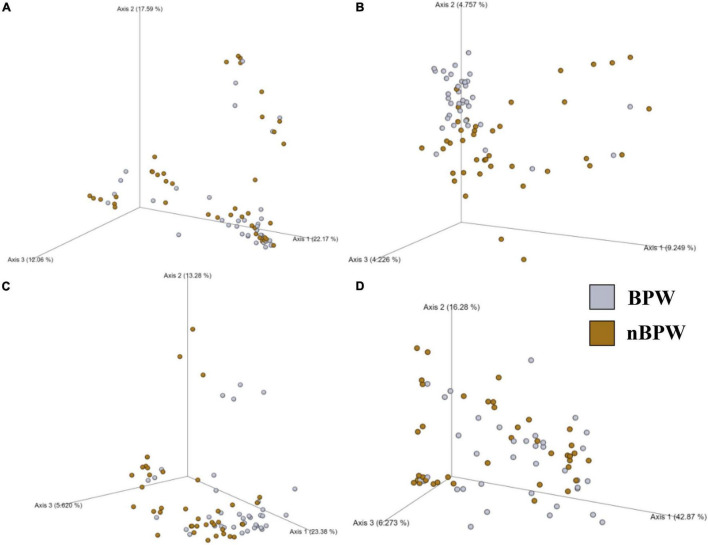
Significant effect of buffer type, nBPW and BPW, of WBCR on the beta diversity metrics: **(A)** Bray–Curtis, **(B)** Jaccard Dissimilarity, **(C)** Unweighted UniFrac, and **(D)** Weighted UniFrac of the original rinsates. Main effects of treatment were determined by using PERMANOVA with a significance level of *P* ≤ 0.05 and *Q* ≤ 0.05 (*N* = 77, *n* = 40 and 37, *P* < 0.05, *Q* < 0.05). Beta diversity metrics are represented as a PCOA plot.

There were no differences between the alpha diversity metrics (Observed Features, Shannon’s Entropy, Faith’s PD, and Pielou’s Evenness) between the BPW and nBPW rinsates (*P* > 0.05; *Q* > 0.05; Table 1). Among the beta diversity metrics (Figure 4 and Supplementary Table 1), PERMANOVA indicated differences (*P* < 0.05; *Q* < 0.05) in the phylogenetic (unweighted and weighted UniFrac) and dissimilarity metrics (Bray–Curtis and Jaccard) between the two populations. Additionally, there was an effect of dispersion on the dissimilarity indices (Bray–Curtis and Jaccard dissimilarity) of the two rinsate types, BPW and nBPW (PERMDISP; *P* < 0.05; *Q* < 0.05).

**TABLE 1:** Effect of buffer type, nBPW and BPW, of WBCR on alpha diversity metrics (Observed Features, Shannon, Faith’s Phylogenetic Diversity, and Pielou’s Evenness) of the original samples (rinsate), non-selective and selective media (APC Petrifilm™ and CCPM agar plates), and enrichments (*Salmonella* spp. and *Campylobacter* spp. enrichments).

	Kruskal–Wallis*				

	BPW	nBPW	*P*-value	*Q*-value	*H*-value^1^
**Original rinsate**					
Observed features	24.6811.80	20.6513.73	0.19	0.19	1.73
Shannon’s entropy	3.211.19	2.691.55	0.19	0.19	1.76
Faith’s PD	3.911.66	3.241.64	0.10	0.10	2.63
Pielou’s evenness	0.700.17	0.620.24	0.25	0.25	1.30
*n*	40	37			
**APC petrifilm™**					
Observed features	3.881.25	2.851.27	<0.01	<0.01	13.7
Shannon’s entropy	1.590.56	1.140.63	<0.01	<0.01	10.5
Faith’s PD	1.100.18	1.000.12	<0.01	<0.01	8.65
Pielou’s evenness	0.830.11	0.800.15	0.62	0.62	0.25
*n*	40	40			
**CCPM**					
Observed features	1.000.00	1.030.16	0.36	0.36	0.84
Shannon’s entropy	0.000.00	0.010.12	0.36	0.36	0.84
Faith’s PD	0.970.02	0.970.02	0.36	0.36	0.84
Pielou’s evenness	0.890.10	0.870.10	0.43	0.43	0.61
*n*	32	38			
***Campylobacter* spp. enrichment**					
Observed features	1.220.49	2.180.75	<0.01	<0.01	15.32
Shannon’s entropy	0.180.40	0.910.56	<0.01	<0.01	14.61
Faith’s PD	0.980.04	1.110.12	<0.01	<0.01	16.65
Pielou’s evenness	NA	0.800.15	NA	NA	NA
*n*	32	11			
***Salmonella* spp. enrichment**					
Observed features	13.243.29	12.583.16	0.41	0.41	0.67
Shannon’s entropy	2.340.44	2.220.62	0.40	0.40	0.69
Faith’s PD	1.400.28	1.590.39	0.06	0.06	3.42
Pielou’s evenness	0.600.09	0.600.14	0.13	0.13	2.33
*n*	36	38			

*Main effects were determined by Kruskal–Wallis with pairwise differences being significant with Q ≤ 0.05.

*^1^H value: test statistic for Kruskal–Wallis.*

**FIGURE 5:**
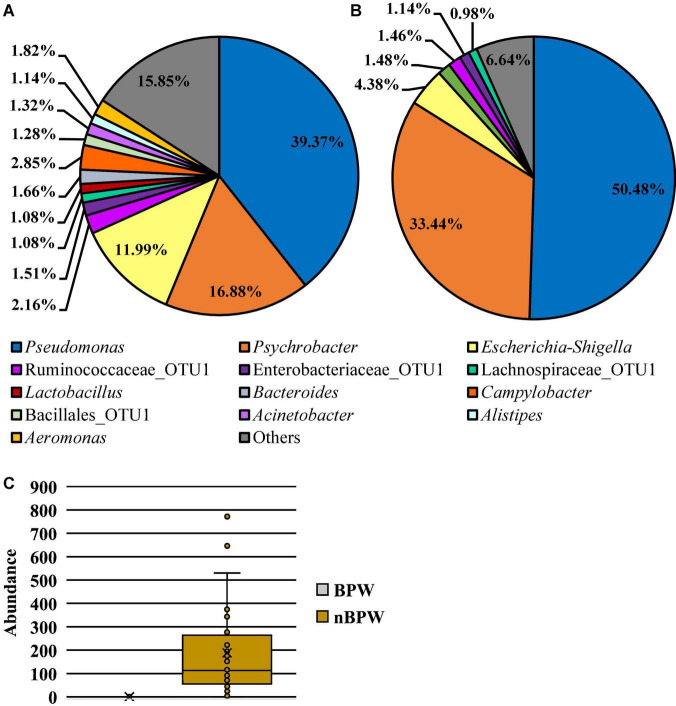
Microbiota compositional profiles at the genus level of the original rinsates from WBCR that used either **(A)** BPW or **(B)** nBPW as the rinsing buffer (*N* = 77, *n* = 40 and 37, *k* = 2). The main effect of treatment (BPW vs. nBPW) was determined using ANCOM, in which only *Clostridium sensu stricto 7* **(C)** was significantly different between the two buffer types, BPW vs. nBPW, used in the collection of the WBCR (*P* < 0.05; *W* = 189). An asterisk (*) denotes significant difference (*P* < 0.05). Low-abundance (<1.0%) OTUs were not labeled to aid in clarity of figures.

Microbiota analysis of the collected rinsates revealed the presence of 15 different OTUs present at relative abundance values greater than or equal to 1% in at least one of the rinsate types, PW or nBPW (Figure 5). The relative abundance values for the observed OTUs in both BPW and nBPW rinsates indicated that the major constituents of both microbiotas belonged to *Pseudomonas*, *Psychrobacter*, and *Escherichia–Shigella*, with those OTUs representing 39.37, 16.88, and 11.99% and 48.70, 32.26, and 4.23% of the microbiota composition, respectively (Figures 5A,B). When using ANCOM to determine the significantly different taxa at the genus level among the two rinsate types, BPW and nBPW, the only significant different was *Clostridium sensu stricto 7* (*W* = 188, *P* < 0.05), belonging to *Clostridiaceae*. *Clostridium sensu stricto 7* was more abundant in the nBPW rinsates than in the BPW rinsates (Figure 5C).

### Impact of Whole Bird Carcass Buffer on the Diversity and Microbiota of Aerobic Bacteria Petrifilm™

**FIGURE 6:**
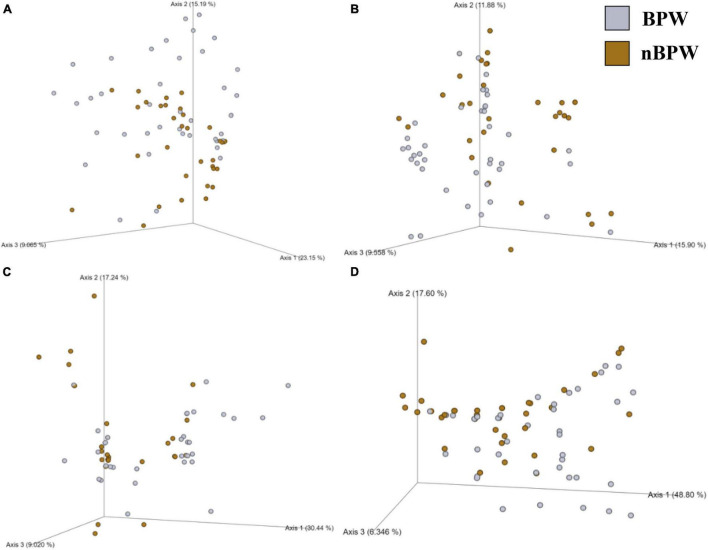
Significant effect of buffer type, nBPW and BPW, of WBCR on the beta diversity metrics: **(A)** Bray–Curtis, **(B)** Jaccard Dissimilarity, **(C)** Unweighted UniFrac, and **(D)** Weighted UniFrac of the non-selective media (APC Petrifilm™). Main effects of treatment were determined by using PERMANOVA with a significance level of *P* ≤ 0.05 and *Q* ≤ 0.05 (*N* = 80, *n* = 40, *P* < 0.05, *Q* < 0.05). Beta diversity metrics are represented as a PCOA plot.

Alpha diversity metrics, Observed Features, Shannon Entropy, and Faith’s PD were significantly different (*P* < 0.01) with samples prepared from BPW rinsates being more diverse overall (*P* < 0.05; *Q* < 0.05; Table 1). The evenness (Pielou’s Evenness) of the two populations (BPW and nBPW) was not significantly different (*P* > 0.05), indicating that the diversity was evenly distributed among all samples and differences could not necessarily be attributed to outliers or unique distributions within a few samples. All beta metrics indicated a significant difference (*P* < 0.05; Figure 6 and Supplementary Table 1) in the two populations when using PERMANOVA, and associated PERMDISP analysis indicated that these significant differences were not due to the uneven dispersion of sequences in the samples (*P* > 0.05).

**FIGURE 7:**
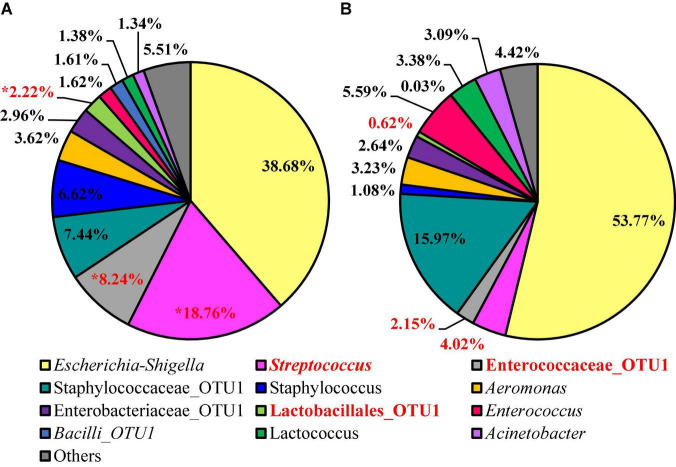
Microbiota compositional profiles at the genus level of the APC Petrifilm™ that had been previously inoculated with 1 ml of WBCR that used either **(A)** BPW or **(B)** nBPW as the rinsing buffer (*N* = 80, *n* = 40, *k* = 2). The main effect of treatment (BPW vs. nBPW) was determined using ANCOM, in in which *Streptococcus*, Lactobacillales, and Enterococcaceae were significantly different between the two buffer types, BPW vs. nBPW, used in the collection of the WBCR (*P* < 0.05; *W* = 60, 59, and 54, respectively). A red asterisk (*) denotes significant difference between treatments (*P* < 0.05). Low-abundance (<1.0%) OTUs were not labeled to aid in clarity of figures.

Twelve different features were identified at relative abundance levels greater than or equal to 1% in the sequences obtained from the 3M™ APC Petrifilm™ at the genus level. *Escherichia–Shigella* was the most prevalent in both populations (38.68 and 53.78%, respectively; Figure 7). *Streptococcus* was the second most abundant feature for BPW (18.76%), and the second most abundant feature for nBPW was Staphylococcaceae (15.98%). Through the use of ANCOM at the genus level, *Streptococcus* (*W* = 59), Enterococcaceae (*W* = 60), and Lactobacillales (*W* = 54) were found to be differentially abundant among the 3M™ APC Petrifilm,™ with those being inoculated with BPW rinsates having a greater abundance of these taxa.

### Minor Effect of Whole Bird Carcass Buffer Type on the Microbiota Recovered From *Campylobacter coli* Chromogenic Plating Medium Agar

**FIGURE 8:**
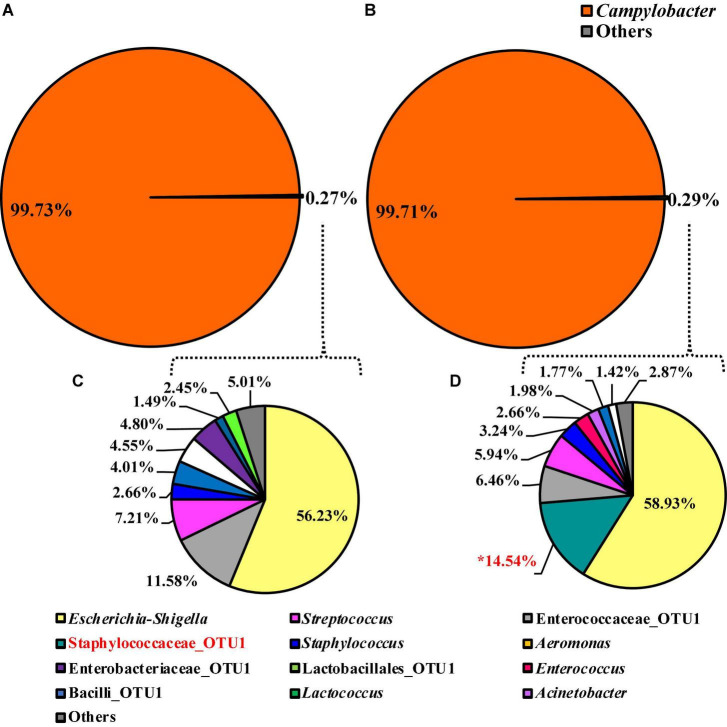
Microbiota compositional profiles at the genus level of the colonies pooled and extracted from the *Campylobacter jejuni*/*C. coli* CCPM agar used to ascertain *Campylobacter* spp. abundance, which had been previously inoculated with 10 μl of WBCR that used either **(A)** BPW or **(B)** nBPW as the rinsing buffer (*N* = 70, *n* = 32 and 38, *k* = 2). The presence of *Campylobacter* OTUs among the feature table was then filtered out to visualize the background microbiota (<0.3% of total population; **C,D**). The main effect of treatment (BPW vs. nBPW) was determined using ANCOM at the genus level prior to any filtering steps, in which Staphylococcaceae were significantly different between the two buffer types, BPW vs. nBPW, used in the collection of the WBCR (*P* < 0.05; *W* = 25). An asterisk (*) denotes significant difference (*P* < 0.05). Low-abundance (<1.0%) OTUs were not labeled to aid in clarity of figures.

The alpha or beta diversity of the microbiota of the colonies pooled, extracted, and sequenced from the *Campylobacter* selective agar plates (CCPM) used to determine the prevalence of *Campylobacte*r spp. was not significantly different (Table 1 and Supplementary Table 1). Overall, 99.73 and 99.71% of the taxa at the genus level were identified as *Campylobacter* within the two rinsate types, BPW and nBPW, respectively (Figure 8). When ANCOM is used, Staphylococcaceae were considered significantly different taxa between the two rinsate types (*W* = 25, *P* < 0.05). Although Staphylococcaceae was significantly different between the two treatment groups, it represented less than 0.30% of the total composition. In order to view the microbiota from 0.3% of the population, *Campylobacter* spp. were filtered out using taxa-based filtering. The resulting composition demonstrates that the colonies from CCPM that had been inoculated with enrichments made from nBPW had a higher relative abundance of Staphylococcaceae than that of BPW (Figure 8D).

### Selective Changes in the Microbiota Diversity and Composition of *Campylobacter* spp. Enrichments Due to Whole Bird Carcass Buffer Type

**FIGURE 9:**
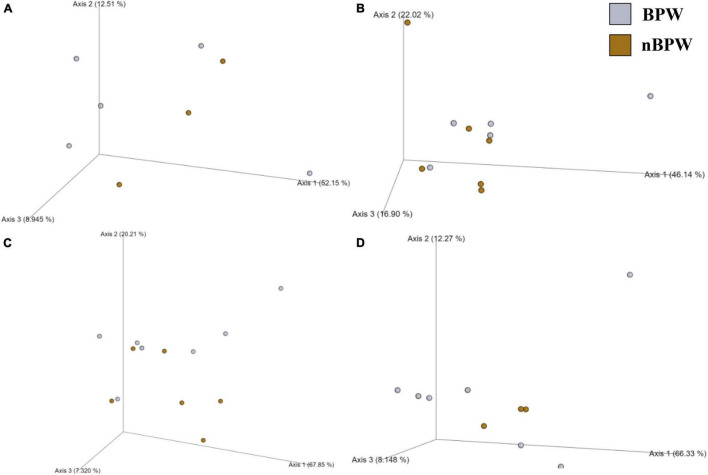
Significant effect of buffer type, nBPW and BPW, of WBCR on the beta diversity metrics: **(A)** Bray–Curtis, **(B)** Jaccard Dissimilarity, **(C)** Unweighted UniFrac, and **(D)** Weighted UniFrac of the *Campylobacter* spp. enrichments. Main effects of treatment were determined by using PERMANOVA with a significance level of *P* ≤ 0.05 and *Q* ≤ 0.05 (*N* = 43, *n* = 32 and 11, *P* < 0.05, *Q* < 0.05). Beta diversity metrics are represented as a PCOA plot.

Unlike the microbiota of the pooled colonies from the CCPM agar plates that had been inoculated with 10 μl of the *Campylobacter* spp. enrichment to determine the prevalence of *Campylobacter* spp., there was a significant difference in the alpha and beta diversity metrics of the *Campylobacter* spp. enrichments from BPW and nBPW rinsates. The Observed Features, Shannon’s Entropy, and Faith’s PD were all higher within the *Campylobacter* spp. enrichments of the nBPW rinsates (*P* < 0.05; *Q* < 0.0.5; Table 1). There was also a difference between the Bray–Curtis, Jaccard dissimilarity, unweighted UniFrac, and Weighted UniFrac of the *Campylobacter* spp. enrichments of the BPW and nBPW rinsates (PERMANOVA; *P* < 0.05; *Q* < 0.05; Figure 9 and Supplementary Table 1). The significance of treatment on the Bray–Curtis, Jaccard dissimilarity, and weighted UniFrac between the *Campylobacter* spp. enrichments of the BPW and nBPW rinsates can be partially attributed to the significant dispersion of the data (PERMDISP; *P* < 0.05; *Q* < 0.05; Figure 9 and Supplementary Table 1). However, there was only a trending significance of dispersion on the unweighted UniFrac between treatments (PERMDISP; *P* = 0.07; *Q* = 0.07).

**FIGURE 10:**
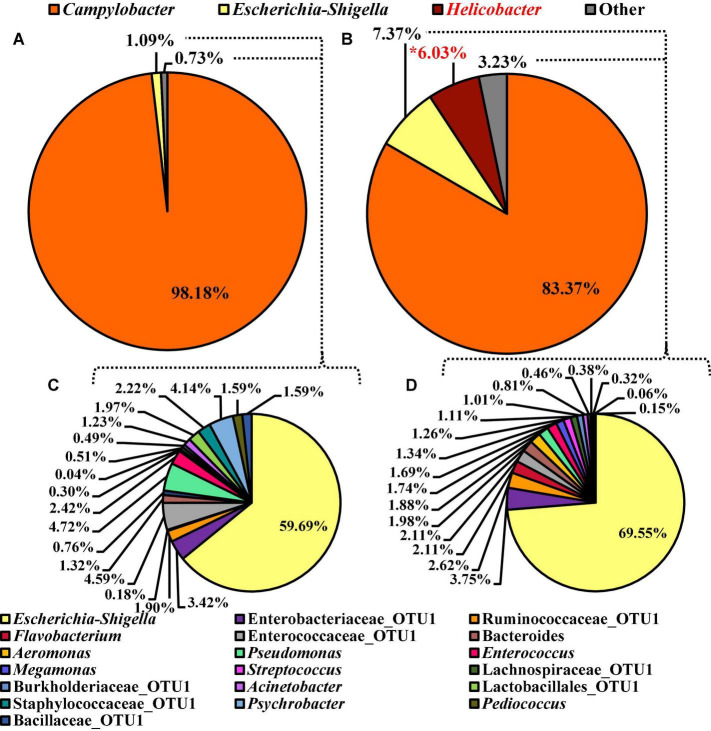
Microbiota compositional profiles at the genus level of the *Campylobacter* spp. enrichments (30 ml rinsate and 30 ml 2 × Bolton’s Broth incubated at 42 ± 1°C for 48 ± 2 h) used to ascertain *Campylobacter* spp. prevalence among WBCR using **(A)** BPW or **(B)** nBPW as the rinsing buffer (*N* = 70, *n* = 32 and 38, *k* = 2). The presence of *Campylobacter* and *Helicobacter* OTUs among the feature table was then filtered out to visualize the background microbiota **(C,D)**. The main effect of treatment (BPW vs. nBPW) was determined using ANCOM at the genus level prior to any filtering steps, in which *Helicobacter* were significantly different between the two buffer types, BPW vs. nBPW, used in the collection of the WBCR (*P* < 0.05; *W* = 57). A red asterisk (*) denotes significant difference (*P* < 0.05). Low-abundance (<1.0%) OTUs were not labeled to aid in clarity of figures.

Unlike the microbiota composition of the pooled colonies from the CCPM agar plates, the *Campylobacter* spp. enrichments exhibited more taxa at the genus level than just *Campylobacter* spp. (Figure 10). Interestingly, *Campylobacter*, *Escherichia–Shigella*, *Helicobacter*, and others represented 98.18, 1.09, 0.00, and 0.73% of the microbiota of *Campylobacter* spp. enrichments of the BPW rinsates (Figure 10A), whereas these populations represented 83.37, 7.37, 6.03, and 3.23% of the total microbiota population (Figure 10B). Of the significantly different abundant taxa at the genus level, as determined using ANCOM, *Helicobacter* was the only significantly different relative abundant taxa comprising 6% of the microbiota of *Campylobacter* spp. enrichments of the nBPW rinsates and 0% of that of the *Campylobacter* spp. enrichments from BPW rinsates (*W* = 57; *P* < 0.05).

To discern the microbiota as *Escherichia–Shigella* and others, *Campylobacter* and *Helicobacter* were filtered out using taxa-based filtering of the feature table. Of the 1.82 and 10.67% relevant abundant taxa that were not *Campylobacter* or *Helicobacter* within the *Campylobacter* spp. enrichments from the BPW and nBPW rinsates, *Escherichia–Shigella* was the highest represented with the other 40 and 30% of the 1.82 and the 10.67% relevant abundant taxa being distributed over 18 different taxa (Figures 10C,D).

### Selective Differences in the Microbiota Diversity and Composition of *Salmonella* spp. Enrichments Due to Whole Bird Carcass Buffer

**FIGURE 11:**
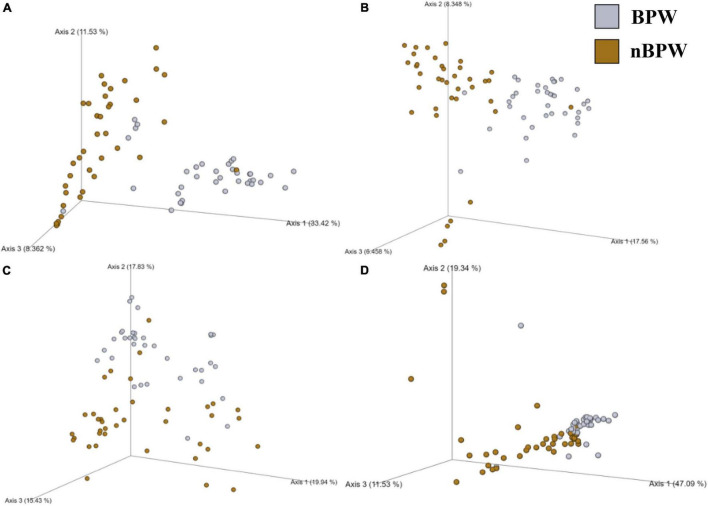
Significant effect of buffer type, nBPW and BPW, of WBC on the beta diversity metrics: **(A)** Bray–Curtis, **(B)** Jaccard Dissimilarity, **(C)** Unweighted UniFrac, and **(D)** Weighted UniFrac of the *Salmonella* spp. enrichments. Main effects of treatment were determined by using PERMANOVA with a significance level of *P* ≤ 0.05 and *Q* ≤ 0.05 (*N* = 74, *n* = 36 and 38, *P* < 0.05, *Q* < 0.05). Beta diversity metrics are represented as a PCOA plot.

There were detected differences within the alpha diversity of the microbiota of the *Salmonella* spp. enrichments from the BPW and nBPW rinsates (*P* > 0.05; *Q* > 0.05; Table 1). However, there was a trending difference between the phylogenetic diversity (Faith’s PD) within the microbiota of the *Salmonella* spp. enrichments from the BPW and nBPW rinsates (*P* = 0.06; *Q* = 0.05; Table 1). As such, the phylogenetic diversity (Faith’s PD) within the microbiota of the *Salmonella* spp. enrichments from the nBPW rinsates (1.59 ± 0.39) tended to be higher than that of the *Salmonella* spp. enrichments from the BPW rinsates (1.40 ± 0.28; *P* = 0.06; *Q* = 0.05). There was a difference between the Bray–Curtis, Jaccard dissimilarity, unweighted UniFrac, and weighted UniFrac of the *Salmonella* spp. enrichments from the BPW and nBPW rinsates (PERMANOVA; *P* < 0.05; *Q* < 0.05; Figure 11 and Supplementary Table 1). The significance of treatment on the Jaccard dissimilarity and the unweighted UniFrac between the *Salmonella* spp. enrichments of the BPW and nBPW rinsates can be partially attributed to the significant dispersion of the data (PERMDISP; *P* < 0.05; *Q* < 0.05; Figure 11 and Supplementary Table 1). However, there was a trending significance of dispersion on the weighted UniFrac between treatments (PERMDISP; *P* = 0.09; *Q* = 0.09).

**FIGURE 12:**
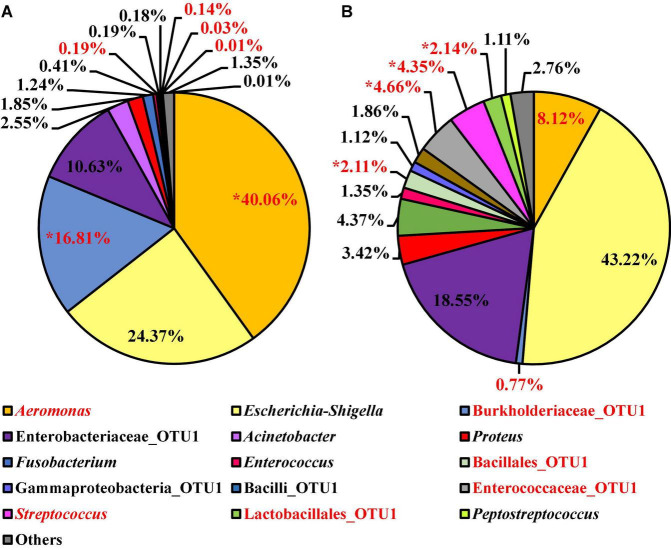
Microbiota compositional profiles at the genus level of the *Salmonella* spp. enrichments (30 ml rinsate and 30 ml BPW incubated at 35 ± 1°C for 20–24 h) used to ascertain *Salmonella* spp. prevalence among WBCR using **(A)** BPW or **(B)** nBPW as the rinsing buffer (*N* = 74, *n* = 36 and 38, *k* = 2). The main effect of treatment (BPW vs. nBPW) was determined using ANCOM at the genus level, in which *Burkholderiaceae*, *Aeromonas*, *Streptococcus*, Enterococcaceae, Lactobacillales, and Bacillales were significantly different between the two buffer types, BPW vs. nBPW, used in the collection of the WBCR (*P* < 0.05; *W* = 55, 54, 53, 53, 51, and 51). A red asterisk (*) denotes significant difference (*P* < 0.05). Low-abundance (<1.0%) OTUs were not labeled to aid in clarity of figures.

At the genus level, 15 OTUs were present at relative abundance levels greater than or equal to 1% in at least one of the *Salmonella* spp. enrichment types (Figure 12). The most abundant taxa within the microbiota of the *Salmonella* spp. enrichments from BPW and nBPW enrichments were *Aeromonas*, *Escherichia–Shigella*, Burkholderiaceae, and Enterobacteriaceae representing 40.06, 24.37, 16.81, and 10.63% of the taxa belonging to *Aeromonas*, representing 40.06% of the microbiota of the *Salmonella* spp. enrichments from BPW and 8.12, 43.33, 0.77, and 18.55% of that of the sequences of *Salmonella* spp. enrichments from nBPW (Figure 12). With ANCOM, the differences between the microbiota of the two *Salmonella* spp. enrichment types were explored at the genus level. Burkholderiaceae, *Aeromonas*, *Streptococcus*, Enterococcaceae, Lactobacillales, and Bacillales were determined to be significantly different taxa among the two *Salmonella* spp. enrichment types (*W* = 55, 54, 53, 53, 51, and 51). Additionally, *Aeromonas* (40.06 and 8.12%) and Burkholderiaceae (16.81 and 0.77%) were significantly more abundant among the microbiota of the *Salmonella* spp. enrichments from BPW than that of the *Salmonella* spp. enrichments from nBPW (*P* < 0.05; Figure 12). However, *Streptococcus* (0.03 and 4.35%), Enterococcaceae (0.14 and 4.66%), Lactobacillales (0.01 and 2.14%), and Bacillales (0.19 and 2.11%) were more abundant among the *Salmonella* spp. enrichments from nBPW than the *Salmonella* spp. enrichments from BPW (*P* < 0.05; Figure 12).

## Discussion

The USDA–FSIS regulatory rule “Pathogen Reduction and Hazard Analysis Critical Control Point” (PR-HACCP), established in 1996, outlined poultry carcass sampling plans which were used to determine the overall prevalence of *Salmonella* in post-chill broilers (USDA-FSIS, 1996). Additionally, the initial data collections were used to establish baseline numbers so that future sampling plans could be used to verify a system’s capabilities in terms of pathogen control. In 2011, USDA–FSIS also began concurrently analyzing samples for prevalence of *Campylobacter* spp. The application of this final rule was in response to increased cases of campylobacteriosis and the need to evaluate commercial processing systems for levels of *Campylobacter* spp. as well as determine an applied system’s ability to reduce and control *Campylobacter* spp. (USDA-FSIS, 2010).

As more stringent standards were placed on processors to reduce both *Salmonella* spp. and *Campylobacter* spp. levels in poultry carcasses and parts, the implementation of novel intervention systems and antimicrobials became standard practice (Smith et al., 2015). Some changes included the addition of a post-chill chemical spray or dip, with a short dwell time as well as antimicrobial solutions with different modes of actions and chemical properties not previously applied to poultry processing systems (Smith et al., 2015; Gamble et al., 2016). The changes in intervention systems and chemical applications introduced opportunities to further evaluate the materials and methods used to perform sample collections in commercial poultry processing. In the case of WBCRs, the use of a BPW rinsate had been used to aid in the recovery of *Salmonella* from collected samples, as it provided a non-selective nutrient source with a built-in phosphate buffering system which helped to maintain neutral pH levels (Baylis et al., 2000). However, as the industry had evolved and introduced new interventions and post-chill applications, Gamble et al. (2016) demonstrated that use of BPW alone may not be sufficient to mitigate antimicrobial carryover that may occur during WBCR sample collection. Furthermore, the presence of a non-neutralized antimicrobial in the rinsate could hinder the recovery of some indicator and pathogenic microorganisms, namely, *Salmonella* from the subsequent rinsates. Thus, the carryover has the potential to lead to false-negative results and hide underlying issues in pathogen control during poultry processing and manufacturing (Gamble et al., 2016).

Therefore, this study aimed at elucidating the downstream impacts of buffer type, BPW and nBPW, on the diversity and composition of the microbiota of WBCRs and their corresponding indicator (APC and EB) and pathogenic (*Campylobacter* and *Salmonella* spp.) microorganisms recovered from traditional, industry-specific, culturing methodologies. As such, the diversity and microbiota composition of BPW and nBPW rinsates that were collected from WBC after the carcasses exited a finishing chiller equipped with PAA at a commercial broiler processing facility and corresponding indicator (3M™ APC Petrifilm™) and pathogenic (*Campylobacter* CCPM agar plates, and *Salmonella* spp. and *Campylobacter* spp. enrichments) culturing matrices were determined. Elucidating the microbiota compositions of the different WBCRs and their corresponding indicator (APC) and pathogenic (*Campylobacter* and *Salmonella* spp.) microorganisms from associated culturing methodologies provides an improved understanding of how rinsate buffer may impact the results of microbiological analyses.

### Non-selective Media Is Selective in Response to the Whole Bird Carcass Buffer Type Used

Bacterial growth from non-selective media, APC and EB Petrifilm™, in the current study is closely aligned with previous work at similar facilities within the same region in Arkansas, United States (Handley et al., 2018). Despite the different pre-enrichments used (Butterfield’s phosphate vs. BPW and nBPW), rinsate samples from WBC exiting the post-chiller had relatively low APC and EB counts (∼1 and < 0.2 log_10_ CFU/ml; Handley et al., 2018). The indicator organisms, APC and EB, were slightly higher in the current study (<0.5 log_10_ CFU/ml difference). This difference could potentially be due to the age of the birds processed (∼4 weeks), as the processing facility was a Cornish facility.

Regardless, it has been established that the use of APC Petrifilm™ is to determine the abundance of indicator organisms within the WBCRs. The APC Petrifilm™ is referred to as non-selective media and is used to promote the growth of APC. However, it has been previously established that the APC Petrifilm™ is somewhat selective and is not completely indicative of the WBCR it was generated from Kim et al. (2017). Kim et al. (2017) demonstrated that the WBCRs using BPW collected after the post-chiller containing PAA were primarily composed of *Paenibacillaceae*, *Bacillus*, and *Clostridium* when examined at the genus level (∼60% of microbiota). However, the corresponding microbiota of the APC Petrifilm™ was heavily composed of *Lysinibacillus* and Planococcaceae (Kim et al., 2017). Although the composition of the WBCRs and the corresponding APC Petrifilm™ in the current study was different than that of Kim et al. (2017), a difference in the composition of the microbiota of the original rinsates and the corresponding APC Petrifilm™ was observed in the current study.

In the current study, *Pseudomonas*, *Psychrobacter*, and *Escherichia–Shigella* were among the most represented taxa at the genus level recovered from the WBCRs, but only *Clostridium sensu stricto 7* was significantly different between the two rinsate buffer types, BPW and nBPW. However, the corresponding APC Petrifilm™ in the current study was primarily composed of *Escherichia–Shigella*, *Streptococcus*, Enterococcaceae, and Staphylococcaceae (∼70%). The relative abundance values of *Streptococcus*, Enterococcaceae, and Lactobacillales among the microbiota of APC Petrifilm™ were significantly different between the two rinse types, BPW and nBPW, with these microorganisms being more abundant within the APC Petrifilm™ of BPW rinsates.

In fact, the microbiota composition of the APC Petrifilm™ did not include the major spoilage indicators identified with aerobic spoilage such as *Pseudomonas* and *Psychrobacter*, which were present within the original rinsates of the BPW and nBPW rinses (Mead, 2004). The microbiota composition of both the original rinsates and the subsequent APC Petrifilm™ did include *Acinetobacter*, another common spoilage organism, at low levels regardless of buffer type used, BPW and nBPW (Mead, 2004). Regarding spoilage, it does appear from the data within this study that the use of nBPW as a WBC rinse may allow greater isolation of spoilage indicator organisms such as *Pseudomonas* and *Psychrobacter*. Being able to accurately define the levels of spoilage microorganisms is becoming increasingly important as integrators find ways to minimize spoilage and contamination. However, these findings will need to be further validated using microbiological and molecular techniques such as quantitative PCR to determine the absolute concentration of the spoilage indicators.

### Acid-Tolerant Microorganisms’ Response to Whole Bird Carcass Buffer Type, Buffered Peptone Water vs. Neutralizing Buffered Peptone Water

As previously mentioned, the microbiota composition of the APC Petrifilm™ is not accurately indicative of the composition of the microorganisms within the original rinsates. Although their compositional profiles differ, the microbiota composition of the APC Petrifilm™ may demonstrate the consequence of using BPW as a WBC rinse. The populations of acid-tolerant organisms were less abundant among the WBCRs, but due to the resuscitative nature of APC Petrifilm™, these acid-tolerant organisms (pH < 7) were more abundant among the APC Petrifilm™, especially the APC Petrifilm™ of the BPW rinsates. *Streptococcus*, Enterococcaceae, and Lactobacillales were among the acid-tolerant organisms that were significantly higher among the APC Petrifilm™ of the BPW rinsates. Therefore, the acid-tolerant organisms comprised a greater proportion of the APC Petrifilm™ prepared from the BPW rinsates compared to those prepared from the nBPW rinsates. This difference in composition most likely impacted the recovery of APC from the APC Petrifilm™ as the BPW rinsates had a higher abundance of total APC (1.60 log_10_ CFU/ml as compared to 1.21 log_10_ CFU/ml).

After the implementation of nBPW by USDA–FSIS in 2016, it became apparent that microbiological methods that use 3M™ Petrifilm™ or other direct plating materials may have been unintentionally affected by the rinse solution due to the initial pH of the rinsates with the pH of nBPW being higher (7.7 ± 0.5 pH at 25°C) than that of BPW (7.2 ± 0.2 pH at 25°C) solutions (Anonymous, 1996, 2018). Microbiota analysis and APC indicator counts (total APC) of this sample set do suggest that the pH level of the rinse solution may alter the recovery of pH-sensitive microorganisms due to low pH levels of the subsequent rinsates (Fricker, 1987). In this specific study, when using pH indicator strips, the pH ranged between 6 and 6.5 for the BPW rinsates and between 7 and 7.5 for the nBPW rinsates. Therefore, the use of nBPW maintained a neutral pH level and may have provided a more robust buffering capacity.

The buffering capacity of these WBCR solutions or buffers is especially important when evaluating intervention strategies using acid or acid blends within poultry processing systems, in which buffering the pH of WBCRs at this step is critical to ensure accurate microorganism recovery and subsequent quantification. Although the USDA–FSIS has recommended the use of nBPW as an acid neutralizer, the buffering capacity in regard to pH can be achieved within BPW WBCR by adjusting the pH level between 6.6 and 7.2 after WBCR collection with the addition of sodium hydroxide (increase pH) or hydrochloric acid (decrease pH) (Anonymous, 2020). In addition, if a facility is using only acid-based interventions, extending the drip time (Gamble et al., 2016) or allowing for a 1 min drip time prior to rinsing the carcass (USDA-FSIS, 2021) may prove to be an effective neutralization step prior to rinsing the WBC.

Furthermore, while the built-in buffering capacity of the nBPW solution is convenient, since the initial starting pH of the solution used is slightly higher than the BPW, the use of nBPW solutions may still require pH adjustment to reach neutral ranges. Adjustment to the pH of the nBPW WBC rinse would be necessary at WBC collection sites prior to the application of acids and for culturing methodology that is pH sensitive such as specific Petrifilm™ or direct plating methodologies. In fact, sample collections by industry and research personnel who may be collecting WBCRs to determine sanitary dress indications, perform process control evaluations, or verify HACCP programs may not always need to use nBPW for all collection sites, and in some cases, the use of nBPW for these collections could add an extra pH neutralization step.

### Selectivity Concerns Regarding Whole Bird Carcass Buffer Type on *Campylobacter* and *Salmonella* spp. Detection

Review of industry-wide *Salmonella* spp. and *Campylobacter* spp. prevalence trends of broiler carcasses after the implementation of nBPW identified a significant (*P* < 0.05) proportional increase (0.02–0.08) in *Salmonella*-positive samples within the commercial broiler processing sector, while the *Campylobacter* spp. prevalence levels in carcasses remained constant at 0.027 (Williams et al., 2018). The increase in *Salmonella* spp. prevalence provided meaningful data in support of the theory that ineffective neutralization of antimicrobials in collected rinsates may have resulted in lower detection rates of *Salmonella* spp. in some samples. These data also highlighted the need to further evaluate the impact of rinsate solution (buffer) on the recovery of *Campylobacter* spp. and their prevalence after effective neutralization of the residual sanitizer (Williams et al., 2018).

While the initial implementation of nBPW by the USDA–FSIS in 2016 did not specifically address whether the results in *Campylobacter* spp. prevalence would be affected (Gamble et al., 2016, 2017), the continued concern of campylobacteriosis in humans and the implication of poultry products as a primary source of this disease highlight the need for accurate estimates of *Campylobacter* on poultry carcasses and parts. Accordingly, there is a need to continue to study the collection procedures and methodologies to evaluate *Campylobacter* spp. levels on WBC collected from commercial broiler processing systems, to ensure that the methods and materials are adequate for recovery of *Campylobacter* spp. (Berrang et al., 2018). In research performed by Bourassa et al. (2019), in which a multi-hurdle poultry process system with PAA interventions was evaluated from pre-chill to post-finishing-chiller using both rinse solutions, overall prevalence levels of *Campylobacter* spp. within the processing system were lower in nBPW rinsates than in BPW rinsates, with nBPW rinsates being only 55% positive while BPW rinsates being 70% positive. In contrast, the current study demonstrated an increase in *Campylobacter* spp. prevalence among the nBPW rinsates, with the nBPW *Campylobacter* spp. enrichments being confirmed as 95% positive, whereas the BPW *Campylobacter* spp. enrichments were only 77.5% positive (*P* = 0.023). Additionally, the compositional profiles of the pooled colonies from the CCPM agar were almost identical with 99.7% of the composition being composed of *Campylobacter* at the genus level. Only the relative abundance of Staphylococcaceae was significant, and it constituted less than 0.30% of the total composition (*P* < 0.05). However, when reviewing the composition of the *Campylobacter* spp. enrichments, the relative abundance of *Campylobacter* was numerically different between the two *Campylobacter* spp. enrichment types, BPW and nBPW, with *Campylobacter* comprising 98.18% of the BPW enrichments and 83.37% of the nBPW enrichments (*P* < 0.05). The only significant change in the composition of the *Campylobacter* spp. enrichments was the relative abundance of *Helicobacter*, which was more abundant among the nBPW enrichments (0.00 and 6.03%; *P* < 0.05).

Enterobacteriaceae-assigned OTUs, specifically *Escherichia–Shigella* OTUs, can be indicative of the presence of *Salmonella* spp. in these sample types because resolution of *Salmonella* sequences in a complex microbial community may be hindered due to the low-level presence of the target sequences coupled with the limitations of identifications based off amplicons produced from one variable region of 16S sequencing (Grim et al., 2017). Additionally, some bioinformatic pipelines may not be able to provide complete resolution of all 16S rDNA sequences, especially for those close phylogenetically related genera (Ceuppens et al., 2017; Grim et al., 2017). Nevertheless, using OTUs representing Enterobacteriaceae, specifically the *Escherichia–Shigella* OTUs, provides valuable information about closely related *Salmonella* spp. in both non-enriched and enriched microbiological sample collections.

Interestingly, there was a numerically higher relative abundance of *Escherichia–Shigella* OTUs (43.22%; *P* > 0.05) in nBPW enrichments than in BPW enrichments (24.37%). Additionally, there was a trending increase (*P* = 0.0736) in the prevalence of *Salmonella* spp. among the nBPW *Salmonella* spp. enrichments (60% positive) compared to those of the BPW *Salmonella* enrichments (40% positive). Despite the lack of statistical significance (*P* > 0.05), these differences in the relative abundance of *Escherichia–Shigella* and prevalence of *Salmonella* spp. may indicate the improved recovery of *Salmonella* spp. through the use of nBPW as the rinse buffer for WBCR collection. Although there was no difference in the total abundance or prevalence of Enterobacteriaceae (log_10_ CFU/ml) between the two rinsates types, through the use of 16S sequencing of the *Salmonella* spp. enrichments, it was determined that nBPW aids in the recovery of *Salmonella*-like spp., including other enteric microorganisms, such as *Enterobacteriaceae*_OTU1 (10.63 and 18.55%; *P* > 0.05) and *Enterococcaceae*_OTU1 (0.14 and 4.66%; ANCOM; *P* < 0.05). Coinciding with the higher abundance of enteric microorganisms present in the nBPW enrichments, there was a greater proportion of non-enteric OTUs belonging to Burkholderiaceae (16.81 and 0.77%; *P* < 0.05) and *Aeromonas* (40.06 and 8.12%; *P* < 0.05) in the BPW enrichments, which are not typically indicative of enteric or fecal contamination associated with processing poultry.

## Conclusion

The microbiota compositions of the WBCR and associated materials from culturing methodologies were directly impacted due to the buffer solutions used to prepare the WBCR, BPW and nBPW. The buffer types used in the current study, BPW and nBPW, continue to be used interchangeably in the industry with limited understanding of the microbiological implications. Therefore, the results of the current study demonstrated specific compositional shifts in response to the buffer type used to collect WBCR after WBC exits a finishing chiller with PAA.

It is generally understood that to ensure that the samples collected from a poultry processing system are a true reflection of the microbial loads present, including sublethal injured microorganisms, it is necessary to either perform carcass collections using a resuscitative medium or supplement rinsates with a resuscitative medium prior to microbiological evaluations. Up to this point, most of the research in understanding how to best recover sublethally injured cells in WBCRs has been focused on recovery of *Salmonella* spp. While introducing materials and methods in sample collections to aid in the recovery of injured *Salmonella* spp. it is necessary to ensure that informative data are obtained for regulatory entities, industry, and research personnel. The failure to ensure that the materials and methods are adequate to aid in recovery of other target pathogens, namely, *Campylobacter* spp., as well as indicator microorganisms, including APC and EB, may result in skewed data not completely indicative of the system being analyzed. Therefore, it may be advantageous for processors, regulatory bodies, and researchers to use both rinsing agents simultaneously to broaden the recovery of sublethal injured microorganism in order to reduce the bias in the recovery of these microorganisms due to the rinsing agent utilized.

Additionally, as the current research demonstrated differences between the microbiota recovered from both original rinses and subsequent microbiological culturing analyses, future studies should be conducted to determine the impact of these rinsing agents, BPW and nBPW, on the isolation of *Salmonella* and *Campylobacter* spp. recovered from poultry carcasses. Determining the specific *Salmonella* and *Campylobacter* serovars and strains recovered from the different rinsing agents will help regulatory bodies to properly report the top serovars of *Salmonella* and *Campylobacter* contributing to significant human foodborne illness. In congruence with Cox et al. (2019), it is expected that these different pre-enrichment rinse agents will directly impact surveillance data and impact both *Salmonella* and *Campylobacter* population dynamics.

Furthermore, the application of one rinse material to all processors, all sampling locations, without consideration for the intervention chemistry being used or the endpoint use of data (fecal contamination determination vs. spoilage potential) could introduce conflicting results between processors, regulatory bodies, and researchers. Understanding the impact of the rinse material on downstream microbiological analysis can help decision makers determine what rinse material to use depending on the stage of the process being evaluated, the intervention chemistries used, and the microbiological results of interest for that sample set. When it is recognized that all rinse materials are not the same, informed decisions can be made as to what material to use, choosing what works best to meet the identified needs.

## Data Availability Statement

The datasets presented in this study can be found in online repositories. The names of the repository/repositories and accession number(s) can be found below: https://www.ncbi.nlm.nih.gov/, PRJNA765223.

## Ethics Statement

Ethical review and approval was not required for the animal study because samples were collected from chicken carcasses post-harvest at a commercial processing facility and therefore were exempt from animal ethics committee.

## Author Contributions

JW, KF, and SR designed the project. JW collected the samples in the current study, performed the microbiological assays, and wrote the initial manuscript. DD performed the 16S rDNA library preparation and sequencing, edited the manuscript, and formatted the final figures and tables. JW and DD analyzed the microbiological and microbiome data. All authors significantly contributed to the work of the manuscript and reviewed the manuscript prior to submission.

## Conflict of Interest

JW is employed by the company Tyson Foods (Springdale, AR, United States). The remaining authors declare that the research was conducted in the absence of any commercial or financial relationships that could be construed as a potential conflict of interest.

## Publisher’s Note

All claims expressed in this article are solely those of the authors and do not necessarily represent those of their affiliated organizations, or those of the publisher, the editors and the reviewers. Any product that may be evaluated in this article, or claim that may be made by its manufacturer, is not guaranteed or endorsed by the publisher.
