# Supplementary material for: Consequences of Implementing Neutralizing Buffered Peptone Water in Commercial Poultry Processing on the Microbiota of Whole Bird Carcass Rinses and the Subsequent Microbiological Analyses
Source: Front Microbiol. 2022 Mar 17;13:813461. doi: 10.3389/fmicb.2022.813461 (PMC8969756; doi:10.3389/fmicb.2022.813461)
Supplement: Supplementary file 1 [file Table_1.docx]

**Supplemental Information**

**Supplemental Table 1 |** Effect of buffer type, nBPW and BPW, of whole bird carcass rinses (WBCR) on the beta diversity metrics (Bray-Curtis, Jaccard Dissimilarity, Unweighted UniFrac, Weighted UniFrac) of the original samples (rinsate), non-selective and selective media (APC Petrifilm^™^ and CCPM agar plates), and enrichments (*Campylobacter* spp. and *Salmonella* spp. enrichments).

|  |  | PERMANOVA^1^ | | | | | | | PERMIDSP^2^ | | | | | | | | |  |  |
| --- | --- | --- | --- | --- | --- | --- | --- | --- | --- | --- | --- | --- | --- | --- | --- | --- | --- | --- | --- |
|  |  | pseudo-F | P-value | | Q-value | | N | | | F-value | | P-value | | Q-value | | N | | |  |
| Rinsate | |  | |  | |  | |  | | |  | |  | |  | |  | | |
|  | Bray-Curtis | 3.16 | < 0.01 | | < 0.01 | | 77 | | | 5.30 | | 0.02 | | 0.02 | | 77 | | |  |
|  | Jaccard Dissimilarity | 2.6 | < 0.01 | | < 0.01 | | 77 | | | 6.42 | | 0.01 | | 0.01 | | 77 | | |  |
|  | Unweighted UniFrac | 3.54 | < 0.01 | | < 0.01 | | 77 | | | 2.04 | | 0.14 | | 0.14 | | 77 | | |  |
|  | Weighted UniFrac | 3.91 | < 0.05 | | < 0.05 | | 77 | | | 0.05 | | 0.82 | | 0.82 | | 77 | | |  |
| APC Petrifilm™ | |  | |  | |  | |  | | |  | |  | |  | |  | | |
|  | Bray-Curtis | 5.04 | < 0.01 | | < 0.01 | | 80 | | | 1.86 | | 0.19 | | 0.19 | | 80 | | |  |
|  | Jaccard Dissimilarity | 5.91 | < 0.01 | | < 0.01 | | 80 | | | 11.84 | | < 0.01 | | <0.01 | | 80 | | |  |
|  | Unweighted UniFrac | 7.08 | < 0.01 | | < 0.01 | | 80 | | | 0.10 | | 0.42 | | 0.42 | | 80 | | |  |
|  | Weighted UniFrac | 7.87 | < 0.01 | | < 0.01 | | 80 | | | 0.43 | | 0.52 | | 0.52 | | 80 | | |  |
| CCPM Extractions | |  | |  | |  | |  | | |  | |  | |  | |  | | |
|  | Bray-Curtis | 0.84 | 1 | | 1 | | 70 | | | 0.84 | | 0.62 | | 0.62 | | 70 | | |  |
|  | Jaccard Dissimilarity | 0.84 | 1 | | 1 | | 70 | | | 0.84 | | 0.96 | | 0.96 | | 70 | | |  |
|  | Unweighted UniFrac | 0.84 | 1 | | 1 | | 70 | | | 0.84 | | 0.73 | | 0.73 | | 70 | | |  |
|  | Weighted UniFrac | 0.84 | 1 | | 1 | | 70 | | | 0.84 | | 0.80 | | 0.80 | | 70 | | |  |
| *Campylobacter* spp. Enrichment | |  | |  | |  | |  | | |  | |  | |  | |  | | |
|  | Bray-Curtis | 4.28 | 0.01 | | 0.01 | | 43 | | | 4.19 | | < 0.05 | | < 0.05 | | 43 | | |  |
|  | Jaccard Dissimilarity | 6.78 | < 0.01 | | < 0.01 | | 43 | | | 10.23 | | < 0.01 | | < 0.01 | | 43 | | |  |
|  | Unweighted UniFrac | 2.63 | 0.03 | | 0.03 | | 43 | | | 3.42 | | 0.07 | | 0.07 | | 43 | | |  |
|  | Weighted UniFrac | 6.78 | < 0.01 | | < 0.01 | | 43 | | | 7.98 | | < 0.01 | | < 0.01 | | 43 | | |  |
| *Salmonella* spp. Enrichment | |  | |  | |  | |  | | |  | |  | |  | |  | | |
|  | Bray-Curtis | 2.19 | < 0.01 | | < 0.01 | | 74 | | | 1.33 | | 0.22 | | 0.22 | | 74 | | |  |
|  | Jaccard Dissimilarity | 11.55 | < 0.01 | | < 0.01 | | 74 | | | 11.84 | | < 0.01 | | <0.01 | | 74 | | |  |
|  | Unweighted UniFrac | 10.57 | < 0.01 | | < 0.01 | | 74 | | | 7.79 | | <0.01 | | < 0.01 | | 74 | | |  |
|  | Weighted UniFrac | 23.49 | < 0.01 | | < 0.01 | | 74 | | | 3.04 | | 0.09 | | 0.09 | | 74 | | |  |

^1^PERMANOVA – a multivariate form of ANOVA with permutations for β diversity, this analysis is used to quantify pairwise comparisons of treatment groups through. The pseudo-F value compares the sum of squares of the between-clusters to that of the within-clusters, with a larger pseudo-F statistic indicating pronounced clustering.

^2^PERMDISP – testing to evaluate if dispersion in OTU reads accounts for the differences in distributions and abundances found in PERMANOVA. The F-value is the ratio of two variances.
